# Expanding and refining the Mammalian Phenotype Ontology to enhance disease model discovery

**DOI:** 10.1242/dmm.052385

**Published:** 2025-10-28

**Authors:** Susan M. Bello, Anna V. Anagnostopoulos, Leigh C. Carmody, Nicolas Matentzoglu, Cynthia L. Smith

**Affiliations:** ^1^ The Jackson Laboratory, Bar Harbor, ME 04609, USA; ^2^ The Jackson Laboratory for Genomic Medicine, Farmington, CT 06032, USA; ^3^ Semanticly, Athens, Attiki 10563, Greece

**Keywords:** Phenotype, Ontology, Disease Model, Comparative Genomics

## Abstract

The mouse is a premier model system for investigating gene function and modeling human disease. For almost 40 years, Mouse Genome Informatics has worked to capture and integrate the data generated from mouse studies. A critical component of this integration is the development and use of the Mammalian Phenotype (MP) Ontology to capture the morphological and physiological effects of alterations to gene function in the mouse. As the wealth of phenotype data captured using the MP has expanded, its utility in the diagnosis of human disease has increased. Tools have been developed to use mouse and human phenotypes in variant identification. To enhance the applicability of the MP in disease diagnosis and increase the ability of researchers to find models for specific research questions, we have undertaken a disease-focused expansion of the MP. In addition, we have worked to improve the alignment of the MP to the Human Phenotype Ontology to make automated translation between mouse and human phenotypes easier and more reliable.

## Teaser

**Summary:** The Mammalian Phenotype Ontology has advanced to enable precise, integrated phenotype descriptions. Mouse Genome Informatics uses the enriched ontology to improve discovery of mouse models of human disease.

## INTRODUCTION

The Mammalian Phenotype (MP) Ontology was originally developed over 20 years ago to enable consistent annotation, retrieval and analysis of phenotypes by both Mouse Genome Informatics (MGI) and Rat Genome Database (RGD) (Smith et al., 2005). All phenotypes in the MP are defined relative to some reference or control. From the initial ∼100 high-level terms covering broad types of phenotypes (e.g. heart/cardiovascular dysmorphology) at its inception, the MP has undergone substantial content expansion and refinement. As of 19 March 2025, the MP contains 14,318 terms falling under 28 high-level categories, with 99.9% of the terms having human readable definitions (https://www.informatics.jax.org/vocab/mp_ontology/). In addition to MGI and RGD, the MP has been adopted for use by the International Mouse Phenotyping Consortium (IMPC) (Groza et al., 2023) and Mouse Phenome Database (MPD) (Bogue et al., 2023), among others, further helping to drive the expansion of MP terms.

Annotations made using the MP are machine readable and have been incorporated into tools used in coding and structural variant analysis and prediction (Smedley et al., 2015; Sánchez-Gaya and Rada-Iglesias, 2023) and widely used in studies evaluating candidate genes for specific human disease or disorders. The tools typically use the Human Phenotype Ontology (HPO) (Gargano et al., 2024) to encode patient and disease phenotype profiles, as the HPO has been widely adopted as a standard for capturing human health data. These tools then use mouse model MP annotations to fill in gaps in human data and to refine and prioritize predictions. MP-based annotations have also been used in a variety of ways related to human disease, including disease candidate gene enrichment analysis following whole-exome sequencing (Lin et al., 2022a,b; Wang et al., 2022), drug target prediction (Hoehndorf et al., 2014), disease model prediction (Cacheiro et al., 2024) and disease gene predictions (Higgins et al., 2022).

Unlike the MP, which is designed to be broadly applicable across all mammals, the HPO is specifically designed to meet the needs of clinicians and researchers describing human phenotypes. This results in differences in both the broad structure of the ontologies and in the labels and definitions of related phenotype terms. For example, the HPO has broad categories, such as ‘Abnormality of head or neck’ (HP:0000152) and ‘Constitutional symptom’ (HP:0025142), that are not represented in the MP. However, descendant terms of these branches often have matching terms in the MP, as exemplified by ‘Long neck’ (HP:0000472) and ‘elongated neck’ (MP:0012720). Labels and definitions in the MP need to apply across all mammals; thus, terms for hands/feet/paws are labeled as autopods and defined as ‘the distal elements of the limb of vertebrates including the pedal or prehensile appendages’ to avoid using species-specific labels or definitions. The HPO includes terms for hand or foot abnormalities but no term that groups hands and feet.

These differences in structure can present challenges for automated translations and comparisons across ontologies. By using common cross-references from external ontologies, shared synonyms and cross-mappings between the ontologies, these challenges can be mitigated. Cross-references to external ontologies are used to point to external terms that are component parts of the phenotype terms. These cross-references are typically in the form of logical definitions, which use standard relations from the Relations Ontology (RO) (https://doi.org/10.5281/zenodo.15790785) to connect terms from other ontologies that encompass the meaning of the term. For example, the MP term ‘increased hindlimb autopod size’ (MP:0000573) is logically defined as ‘increased size and characteristic of some pes and has modifier some abnormal’. Similarly, the HPO term ‘Long foot’ (HP:0001833) uses the species-neutral Uber-anatomy ontology (Uberon) (Mungall et al., 2012) term ‘pes’ (UBERON:0002387) in its logical definition, making it clear that both phenotypes are describing the analogous anatomical structure. During the course of MP development, we focused on maintaining and expanding these connections to the HPO to increase the ability of researchers to apply MP annotated data to translational studies.

## RESULTS

Since the last publication on the development of the MP, the MP has expanded from 11,464 (1 January 2017) to 14,318 terms (19 March 2025). New terms are identified for addition to the MP through a number of routes. In the process of curation of literature or direct data submissions, curators at MGI, RGD, MPD, IMPC, The Jackson Laboratory (JAX) or other users of the MP may identify phenotypes that are not yet represented in the MP and request new terms. Another major source of new terms is the periodic review of annotations within MGI to MP terms with a large number (30 or more) of direct annotations. These reviews identify more granular terms that can be split off the existing term and added to the MP. Further terms are added as part of systematic review of specific branches within the MP hierarchy, such as reproductive system or craniofacial phenotypes (see below). More recently, we have begun disease-focused expansion of the MP. This work has involved both literature-driven term identification and expansion based on HPO term annotations to these human diseases.

### Pediatric disease expansion

The Gabriella Miller Kids First Pediatric Research Program has established the Kids First Data Resource Center (KFDRC) to bring together patient data for pediatric cancer and congenital disorders. The KFDRC integrates these data using HPO terms to standardize patient signs and symptoms and makes the integrated data accessible to researchers worldwide. To facilitate the discovery of relevant models and model organism data for use in advancing the understanding of pediatric diseases and disorders, MGI and the Zebrafish Information Network (ZFIN) initiated focused curation and ontology development for diseases within the Kids First umbrella. These diseases were evaluated to identify those with significant additional uncurated literature in the MGI and ZFIN resources. From these, six diseases or disease categories were selected for intensive review and curation (see Table 1). During curation, phenotypes displayed by mouse models were identified and compared to existing terms within the MP. In cases in which the model phenotypes did not exactly match an existing MP term, the requirement for new MP terms was evaluated. For example, curation of new mouse models of idiopathic scoliosis identified models that displayed a shift in the position of the nucleus pulposus in the curved region of the spine (Karner et al., 2015). Based on this and review of additional annotations, a new term for ‘abnormal nucleus pulposus morphology’ (MP:0006392) was added to the MP.

**Table 1.** Pediatric diseases reviewed, number of papers reviewed/curated, number of new MP terms added

Disease	Papers reviewed (curated)	Phenotype annotations	MP terms added
Idiopathic scoliosis (DOID:0060250)	53 (35)	122	6
Cleft palate (DOID:674)	912 (61)	886	23
CHARGE syndrome (DOID:0050834)	52 (20)	397	29
Cornelia de Lange syndrome (DOID:11725 and descendants)	32 (5)	98	29
Congenital heart disease* (DOID:1682)	362 (40)	577	48
Osteosarcoma (DOID:3347)	129 (8)	11	3
Down syndrome^‡^ (DOID:14250)	nd	nd	2

*The review of congenital heart disease included the descendants of this term in the Disease Ontology (26 subtypes), with curation done for patent foramen ovale, ventricular septal defect, scimitar syndrome, double outlet right ventricle and dextrocardia. The other descendants of this term either had good coverage already in Mouse Genome Informatics (MGI) or no publications involving mouse models in PubMed. ^‡^Down syndrome already had extensive curation in MGI, but review of Human Phenotype Ontology (HPO) terms associated with this disease resulted in the creation of two new Mammalian Phenotype (MP) terms. nd, not done.

The revision of cleft palate models and MP terms was coordinated with work by the HPO team to revise cleft palate terms in the HPO. In addition to expanding the set of terms for specific subtypes of cleft palate, literature curation also necessitated the creation of terms for phenotypes involved in specific aspects of palate development, such as ‘abnormal palatal mesenchymal cell proliferation’ (MP:0021215) and ‘abnormal palatal shelf bone ossification’ (MP:0021217). In general, these terms do not have matching HPO terms, reflecting the ability of the models to delve deeper into mechanism-related phenotypes.

Another avenue for identification of new terms to add to the MP was the review of HPO terms associated with each disease. HPO terms associated with each disease term were extracted from the HPO site and manually mapped to MP terms. Mappings are in the Simple Standard for Sharing Ontological Mappings (SSSOM) format and publicly available in the Mouse-Human Ontology Mapping Initiative (MHMI) GitHub repository (https://github.com/mapping-commons/mh_mapping_initiative). These files include the date of curation and disease associated with the term. When the addition of a new MP is deemed useful, a link to the related MP issue is included in the comment column. For example, the HPO term ‘Delayed skeletal maturation’ (HP:0002750) is associated with several types of Cornelia de Lange syndrome. In the initial review, the closest match in the MP was ‘abnormal skeletal development’. This was mapped as a narrow match, meaning that the HPO term represented a narrower concept than the MP term, and an MP issue was created to indicate that we needed to review annotations and decide whether a new term or terms should be added to the MP. The review considered (1) whether there were existing annotations in MGI that could be moved to the new term(s) and (2) whether the potential new terms were broadly applicable to all mammals or were specific to humans. In this example, three new terms were added: ‘abnormal skeletal maturation’ (MP:0014294), ‘delayed skeletal maturation’ (MP:0014296) and ‘premature skeletal maturation’ (MP:0014295). In addition, the existing term, ‘abnormal ossification involved in bone maturation’ (MP:0011722), was moved to be a child of the new ‘abnormal skeletal maturation’ term.

In addition to extracting specific disease-associated HPO terms, we also mapped HPO terms in use in KFDRC. The set of HPO terms in use by KFDRC as of 27 May 2021 was extracted along with usage counts (number of unique sampleIDs in KFDRC associated with that phenotype). The usage numbers were used to prioritize highly used HPO terms for mapping to the MP. Again, terms without an exact match in the MP were evaluated to determine whether new MP terms should be added. For example, the HPO term ‘Cutaneous mastocytosis’ (HP:0200151) was associated with three Kids First samples, but the closest term in the MP was ‘increased mast cell number’ (MP:0000324). After review, ‘mastocytosis’ was added as an exact synonym for ‘increased mast cell number’, and a new child term, ‘cutaneous mastocytosis’ (MP:0031395), was created. The new MP term has 14 annotations in MGI (as of 9 September 2025).

In total, curation of pediatric disease models resulted in the addition of 140 new MP terms. The greatest expansion of terms arose from models of congenital heart disease, with 48 new terms added. SSSOM mapping of MP and HPO terms included 1146 HPO terms, 1353 MP terms and a total of 1561 mappings. Because the mappings are not all exact, a single MP or HPO term may be mapped to more than one term in the other ontology.

### COVID-19 expansion

During the coronavirus disease 2019 (COVID-19) pandemic, the *bioRxiv* and *medRxiv* COVID-19 preprints were triaged to identify those that either made use of mouse models or described the signs and symptoms of COVID-19 in patients. Papers using mouse models were curated with emphasis placed on identifying novel phenotypes. Phenotypes without an exact match in the MP were evaluated to see whether new terms should be added. Signs and symptoms for patients were collected from the literature and mapped to both MP and HPO terms. The MP and HPO terms were mapped to the descriptions from the literature and to counterpart terms in each ontology. From this process, 68 issues were opened in the MP GitHub repository (labeled with ‘COVID-19’). Sixty-four of these issues have been closed as resolved. The set of signs/symptoms to MP and HPO term mappings is available in the MP GitHub repository. This file includes the reference(s) that are the source for each sign or symptom. The direct MP to HPO mappings are also in the MHMI GitHub repository. These all have ‘COVID’ in the comment column. We identified 352 distinct sign or symptom descriptions in the literature. These were mapped to 299 HPO terms and 282 MP terms. Not all signs or symptoms had a matching term in both ontologies. For example, reports of confusion or brain fog match the HP terms ‘Confusion’ (HP:0001289) and ‘Brain fog’ (HP:0033630) but do not have matching terms in the MP. As both of these phenotypes are highly human specific and unlikely to be described in the context of non-human mammals, these and similar terms are typically not incorporated into the MP. In some cases, a single symptom may match multiple terms in the MP. For example, the symptom ‘fever’ could be mapped to the MP terms ‘increased body temperature’ (MP:0005533), ‘increased body surface temperature’ (MP:0011017) and ‘increased core body temperature’ (MP:0011016). COVID-19-related terms spanned 14 systems in the MP and HP.

**Fig. 1.**
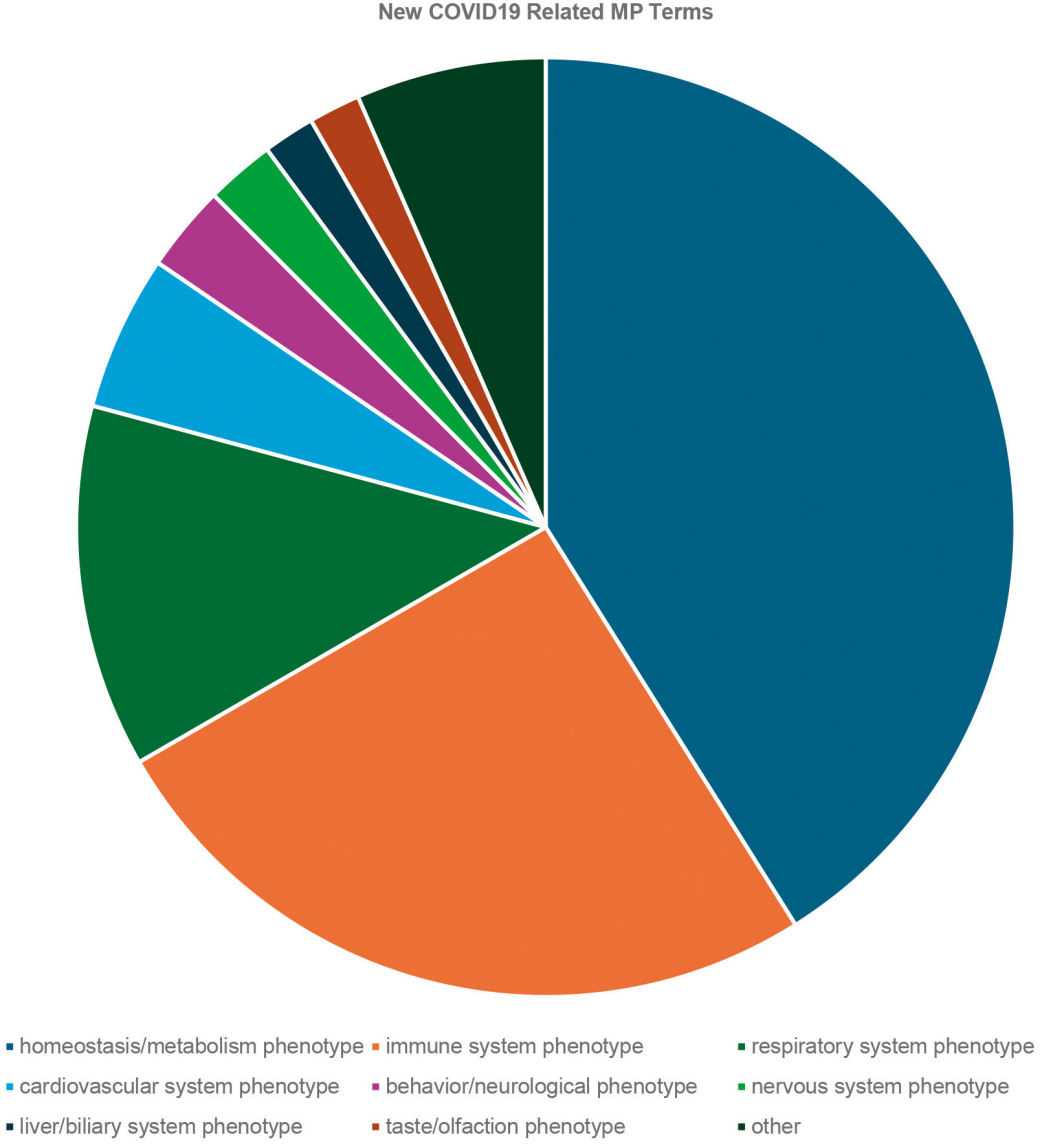
**Breakdown of the new COVID-19-related terms added to the Mammalian Phenotype (MP) Ontology by major systems.** Systems in the figure (count of terms) are as follows: homeostasis/metabolism phenotype (69), immune system phenotype (43), respiratory system phenotype (21), cardiovascular system phenotype (nine), behavior/neurological phenotype (five), nervous system phenotype (four), liver/biliary system phenotype (three), taste/olfaction phenotype (three). Systems with only one or two new MP terms are grouped as ‘other’, which includes cellular (two), growth/size/body region (two), hematopoietic system (two), vision/eye (two), craniofacial (one), integument (one) and muscle (one) phenotypes.

The expansion of the MP to meet the needs of COVID-19 curation resulted in the creation of 124 new MP terms, including terms relevant to severe COVID-19 symptoms such as ‘diffuse alveolar damage’ (MP:0031237), ‘cytokine storm’ (MP:0031066) and ‘decreased blood oxygen saturation level’ (MP:0031068). When adding a term such as ‘decreased blood oxygen saturation level’, we also added ‘abnormal blood oxygen saturation level’ (MP:0031067) as a parent term and ‘increased blood oxygen saturation level’ (MP:0031069) as a sibling class when appropriate. These terms were spread across 15 high-level categories in the MP, with most falling under homeostasis/metabolism or immune system phenotypes (Fig. 1).

**Fig. 2.**
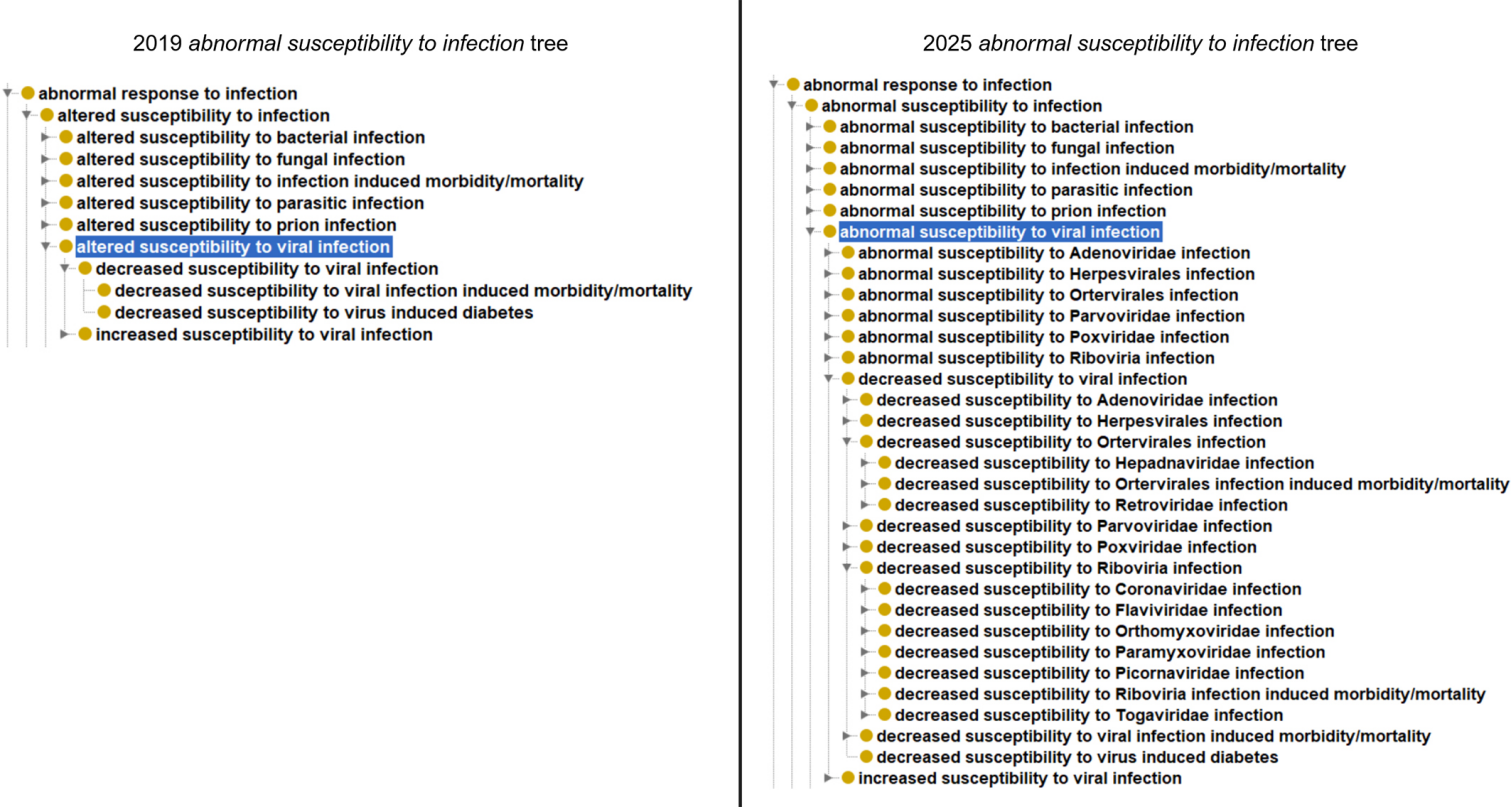
**Comparison of the ‘abnormal susceptibility to viral infection’ branch before (2019) and after (2025) the expansion of this branch during the COVID-19 pandemic.** Terms were added for orders and families of viruses commonly studied in the mouse. Created in BioRender by Bello, S. (2025). https://BioRender.com/ipxlhk5. This figure was sublicensed under CC-BY 4.0 terms.

In addition to creating new terms for phenotypes associated with severe acute respiratory syndrome coronavirus 2 (SARS-CoV-2) infection, we also revised the ‘abnormal susceptibility to viral infection’ hierarchy in the MP. Previously, this branch had grouped all types of viral infection under a single term with only increased and decreased susceptibility descendants. This was revised to add new terms for commonly studied viral orders and families (Fig. 2). Annotations were revised to use the new terms. This makes it easier to find mouse models with alterations in response to specific groups of viruses more readily. For example, the new terms allow a user to discern that mice carrying the allele *Ace2^tm1Pngr^* display decreased susceptibility to Coronaviridae infection and increased susceptibility to Orthomyxoviridae infection in the summary table for this allele at MGI (https://www.informatics.jax.org/allele/MGI:2661723).

### Alzheimer's disease expansion

To improve the breadth and depth of coverage for Alzheimer's disease-related phenotypes, we conducted a review of Online Mendelian Inheritance in Man (OMIM) clinical synopses and published literature for Alzheimer's disease. This review generated 27 GitHub issues for review of MP structure and/or addition of new terms, resulting in the creation of 32 new terms. The new terms included those related to amyloid isoform levels, expansion of the amyloidosis branch with additional location-specific subtypes, and addition of brain lesion and infarct terms. Essentially all the new terms fell under either the nervous system or homeostasis/metabolism phenotype branches. These new MP terms, along with terms associated with annotated mouse models of Alzheimer's disease, were then mapped to HPO terms. This resulted in 22 mappings involving 21 HPO terms and 21 MP terms.

### Domain-focused expansions

**Fig. 3.**
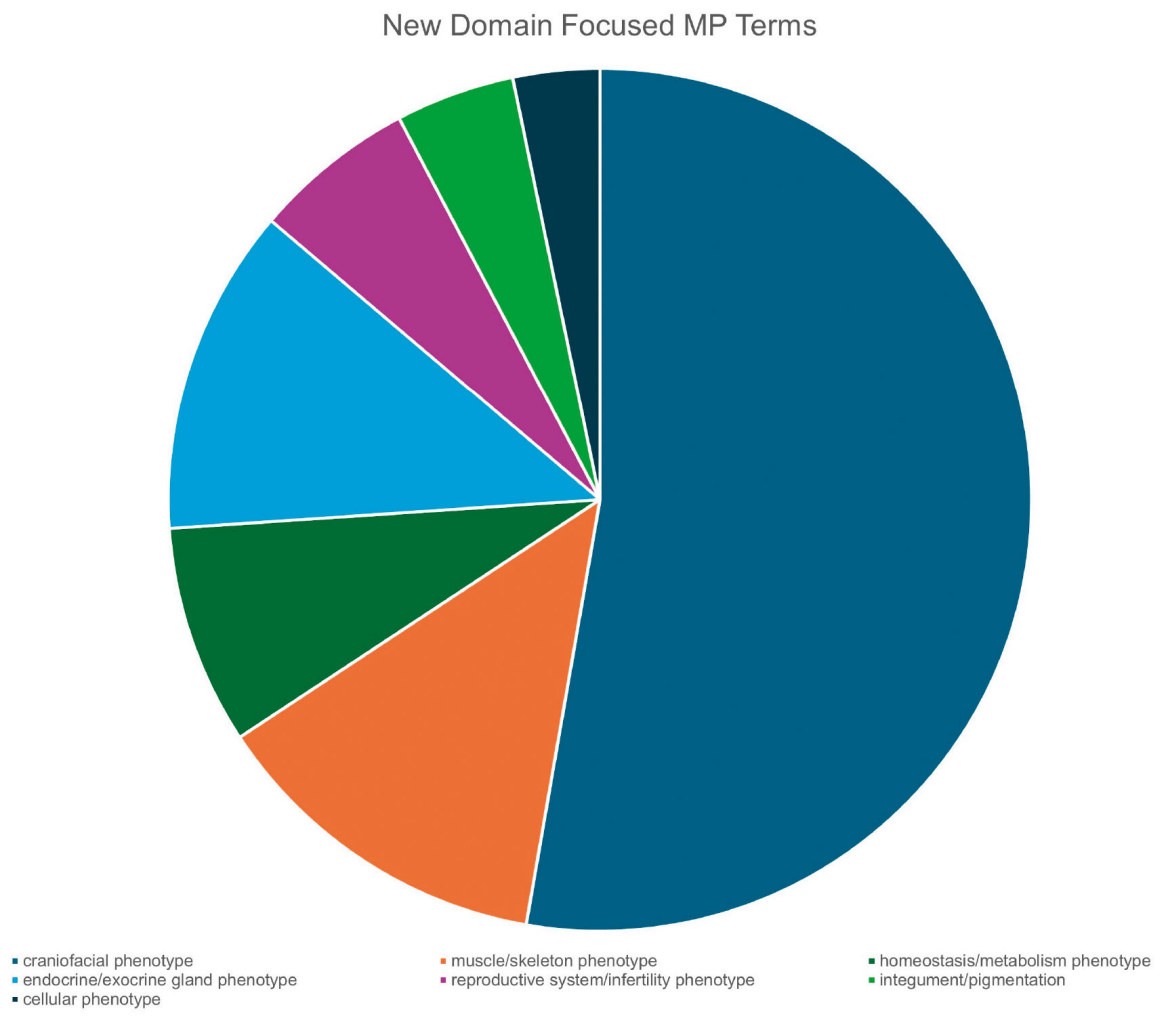
**Breakdown of the new terms added to the MP (2015 to present) created during domain-focused curation by major systems.** Systems in the figure (count of terms) are as follows: craniofacial phenotype (520), muscle/skeleton phenotype (128), homeostasis/metabolism phenotype (81), endocrine/exocrine gland phenotype (121), reproductive system/infertility phenotype (60), integument/pigmentation (44) and cellular (32) phenotype.

Over the past decade, we have systematically evaluated and extended select branches of the MP hierarchy, including endocrine/exocrine glands, craniofacial, homeostasis, reproductive system (with an emphasis on infertility), skeletomuscular system and cellular phenotypes (Smith and Eppig, 2015). These reviews resulted in the addition of ∼1000 new MP terms (Fig. 3). Many of the new terms represent highly specific morphological and physiological phenotypes, such as ‘double-headed sperm’ (MP:0031409) and ‘abnormal intramanchette transport’ (MP:0031387), detected in mouse models. As mouse models often dissect phenotypes seen in human patients to determine developmental and mechanistic origins, the phenotype terms in the MP are frequently more granular than those in the HPO. For example, the term ‘abnormal intramanchette transport’ was explicitly created to enhance annotation of a mouse model of human *SPAG17* deficiency (Kazarian et al., 2018). In comparison, HPO encodes phenotype annotations of human *SPAG17*, orthologous to mouse *Spag17*, at a much coarser level of detail, using terms such as ‘Reduced sperm motility’ (HP:0012207) and ‘Male infertility’ (HP:0003251). The mouse model has 22 annotations that capture details of the precise sperm abnormalities along with other phenotypes.

### Improved alignment with HPO

We have worked with the HPO team to review and revise areas in which the MP and HPO are out of alignment. Enhancing alignment between the two ontologies will make translation between the two ontologies easier and less error prone.

Mappings between MP and HPO terms were analyzed to identify areas for focused improvements in the alignment in several ways. First, during the mapping process, textual definitions were reviewed, and questions were discussed by MP and HPO developers. For example, the HPO had the term ‘Midface retrusion’ (HP:0011800) with the synonym ‘Midface hypoplasia’, while the MP had separate terms for ‘midface retrusion’ (MP:0009855) and ‘midface hypoplasia’ (MP:0012085). After review, the MP term ‘midface retrusion’ was obsoleted and added as an exact synonym to ‘midface hypoplasia’. The term ‘midface hypoplasia’ was retained as the primary label in the MP, as most annotations in MGI used hypoplasia rather than retrusion and, in some cases, used hypoplasia to refer to the mouse phenotype, restricting the use of retrusion to the human phenotype.

Not all of the identified differences could be resolved. In these cases, the mappings serve to warn computational users of differences in meaning that might otherwise be missed. For example, HPO has the term ‘Hypospadias’ (HP:0000047) and MP has the term ‘hypospadia’ (MP:0003124). Based on the term labels, these appear to represent the same phenotype. However, the HPO term is specific to males, whereas the MP term applies to both males and females. In the mapping file, these have the match type ‘skos:narrowMatch’, indicating that the HPO term covers only a subset of the phenotypes covered by the MP term. In MGI, annotations to this term include references to both sexes. When an annotation is specific for a single sex, this information is explicitly added to the annotation in a separate field. As part of the resolution of this issue, we also added terms for types of hypospadias to the MP and moved annotations to these in MGI when possible. For example, we added the term ‘perineal hypospadia’ (MP:0031300) for the type of hypospadia in which in males the urethral opening is located in the perineal region and moved two annotations to this term.

The mappings were further used to increase the consistency of logical definitions in the MP and HPO. Logical definitions can be used to programmatically determine when two terms have the same meaning. The set of MP-HPO terms manually mapped as exact matches but with differing logical definitions were extracted, and these logical definitions were manually reviewed. These differences could be resolved by modifying the MP logical definition to match the HPO one, modifying the HPO definition to match the MP one, modifying both logical definitions or modifying the mapping between the terms. There were 77 term pairs reviewed. Logical definitions for 18 MP terms were updated after this review. The most common change to a logical definition was to change the RO term in the definition from ‘characteristic of’ to ‘characteristic of part of’. In other cases, differences in the choice of anatomical entity were resolved. For example, in the logical definition for anophthalmia (MP:0001293), the anatomical entity was changed from ‘camera-type eye’ (UBERON:0000019) to ‘eyeball of camera-type eye’ (UBERON:0010230) to match the choice in HPO. In addition, mappings for 13 term pairs were modified. Changes suggested for the HPO were submitted to the HPO team.

In addition to reviewing logical definitions for exact matches, we also extracted terms for which the logical definitions were identical in the MP and HPO, but the manual mapping was not exact. There were 25 term pairs in this set. In most cases, the mismatch here was the result of the logical definition lacking nuances present in the textual definition. For example, ‘Polycystic kidney dysplasia’ (HP:0000113) and ‘polycystic kidney’ (MP:0008528) share the same logical definition but are mapped as a narrowMatch. This is due to an incomplete logical definition for the HPO term, which is missing the criteria in the written definition for the cysts to be present in both kidneys. This highlights the difficulty in crafting a logical definition that fully represents the nuances of the meaning of a term. The manual mappings between MP and HPO terms will be useful in refining tools using the MP and HP by resolving some of these issues.

As part of the Human Mouse Disease Connection (HMDC) MP-HPO Match tool (see below), we generated lexical mappings between MP and HPO terms based on term labels and synonyms. Synonym types were used in determining the type of match, e.g. a match between two exact synonyms was called an exactMatch. All terms with more than one exact match to a term in the other ontology were extracted and reviewed to determine whether the synonym type was correct in the ontology. For example, the term ‘decreased bone mineral density’ (MP:0000063) was called an exact match to the terms ‘Osteopenia’ (HP:0000938) and ‘Reduced bone mineral density’ (HP:0004349) based on synonym matches. After review, the synonym type for ‘osteopenia’ was changed from exact to narrow in the MP. These changes are automatically picked up by the match tool when the lexical matches are regenerated.

### Findability, accessibility, interoperability and reusability (FAIR) improvements

To enhance the interoperability of the MP, we have worked to standardize the metadata in the MP to meet the current Open Biological and Biomedical Ontology (OBO) Foundry standards. The OBO Foundry was established to develop a set of interoperable ontologies to support biomedical data integration (Smith et al., 2007). We have updated the basic set of metadata attached to each new term to use ORCID iDs in the contributor field for each person who contributed to the creation or modification of the term. Wherever possible, we have replaced older creator and contributor IDs with ORCID iDs. For example, the contributor MGI:smb has been replaced with orcid.org/0000-0003-4606-0597 throughout the file. New terms now use standard date format xsd:dateTime.

Recently, the OBO Foundry has established a dashboard to report on automated checks on the implementation of OBO principles in ontologies (https://dashboard.obofoundry.org/dashboard/mp/dashboard.html). We have corrected all errors detected by the OBO Dashboard pipeline and reduced the number of warnings reported. Error correction primarily involved updating a small set of relations in the ontology to ensure that they use valid RO terms. Warnings include terms with duplicate synonyms, synonyms that match a term label or missing definitions. The number of MP terms with missing definitions has been reduced from 112 to ten. The remaining ten terms have proven difficult to precisely define, but we continue to work on generating necessary and sufficient definitions. Terms with duplicate exact synonyms all involve terms that share an acronym. For example, the terms ‘decreased prepulse inhibition’ (MP:0009142) and ‘decreased paired-pulse inhibition’ (MP:0014256) both have the exact synonym ‘decreased PPI’ as these share the acronym ‘PPI’. There are now only three sets of terms that share an exact synonym. These updates reduce ambiguity in the file, making it easier for tools to ingest and parse the file.

As part of the Unified Phenotype Ontology (uPheno) project (https://github.com/obophenotype/upheno) (Matentzoglu et al., 2025), standard patterns for logical definitions have been developed for many common types of terms across species. Each pattern consists of constant and variable ontology terms. For example, the ‘abnormalMorphologyOfPartOfAnatomicalEntity’ pattern specifies the variable ‘anatomical entity’, which can be filled with any term from the Uber-anatomy ontology (Uberon). Constants in this pattern include the Phenotype and Trait Ontology (PATO) terms ‘morphology’ (PATO:0000051) and ‘abnormal’ (PATO:0000460). Variables and term IDs are entered in a spreadsheet and stored in the GitHub repository. Patterns also include sections for automatically generating definitions, labels and synonyms, but these are not used in the MP pipeline. The build pipeline removes any existing logical definition and replaces it with the one generated by the pattern. Once incorporated into the ontology build pipeline, the structure of the definitions can be updated for all terms using the pattern by making a change in the pattern file. We have incorporated 32 patterns into the MP build pipeline covering 2793 terms. Prior to adding a new pattern, potential terms fitting that pattern were extracted along with any existing logical definitions. These were reviewed to ensure that the terms fit the pattern and that the correct filler terms were in use.

To increase the recognizability of the MP, we have created a logo for the MP (Fig. S1). This logo is visible at the MP GitHub repository, on the MP OBO Foundry page (https://obofoundry.org/ontology/mp.html) and in the Ontology Lookup Service (OLS) ontology list (http://www.ebi.ac.uk/ols4/ontologies).

### MP internationalization effort

To improve accessibility of the ontology for different communities, we have added alternative British spellings of terms as synonyms throughout the ontology. We have also modified the build pipeline to make use of the translation pipeline developed for the HPO (Gargano et al., 2024). A team at RIKEN provided a Japanese translation file (mp-ja.babelon) for MP term labels (in preparation), and this has been incorporated into the MP pipeline. The file with translations (mp-international) is available with each release and from the MGI ftp site (https://www.informatics.jax.org/downloads/reports/index.html#pheno). This file is in use on the OLS website (https://www.ebi.ac.uk/ols4/ontologies/mp), allowing users to easily switch between English and Japanese versions of the terms. This pipeline will allow for incorporation of additional translation files as they become available.

### Application of MP-HPO mappings in the HMDC

The HMDC search tool at MGI allows users to search for mouse and human phenotype and disease data in a single tool. Users may search using genes, phenotypes, diseases or genomic regions. For genes, phenotypes and diseases, users can search by label or ID. Searching by labels will search across all the ontologies used for annotations. Thus, a search for ‘cleft palate’ will return genes annotated to the MP term ‘cleft palate’, HPO term ‘Cleft palate’ and Disease Ontology (DO) term ‘EEC syndrome’ (based on the synonym ‘ectrodactyly, ectodermal dysplasia, and cleft lip-palate syndrome’; DOID:0060782), among others (Schriml et al., 2019). When searching by phenotype ID, matches can be limited to more specific terms; but, as mouse and human phenotypes are annotated using the MP and HPO, respectively, this requires a user to enter all the corresponding IDs from each ontology. To help users find these counterpart terms, we have added the MP-HP match tool to the HMDC. This tool makes use of both manual and algorithm-derived mappings between the MP and HPO.

**Fig. 4.**
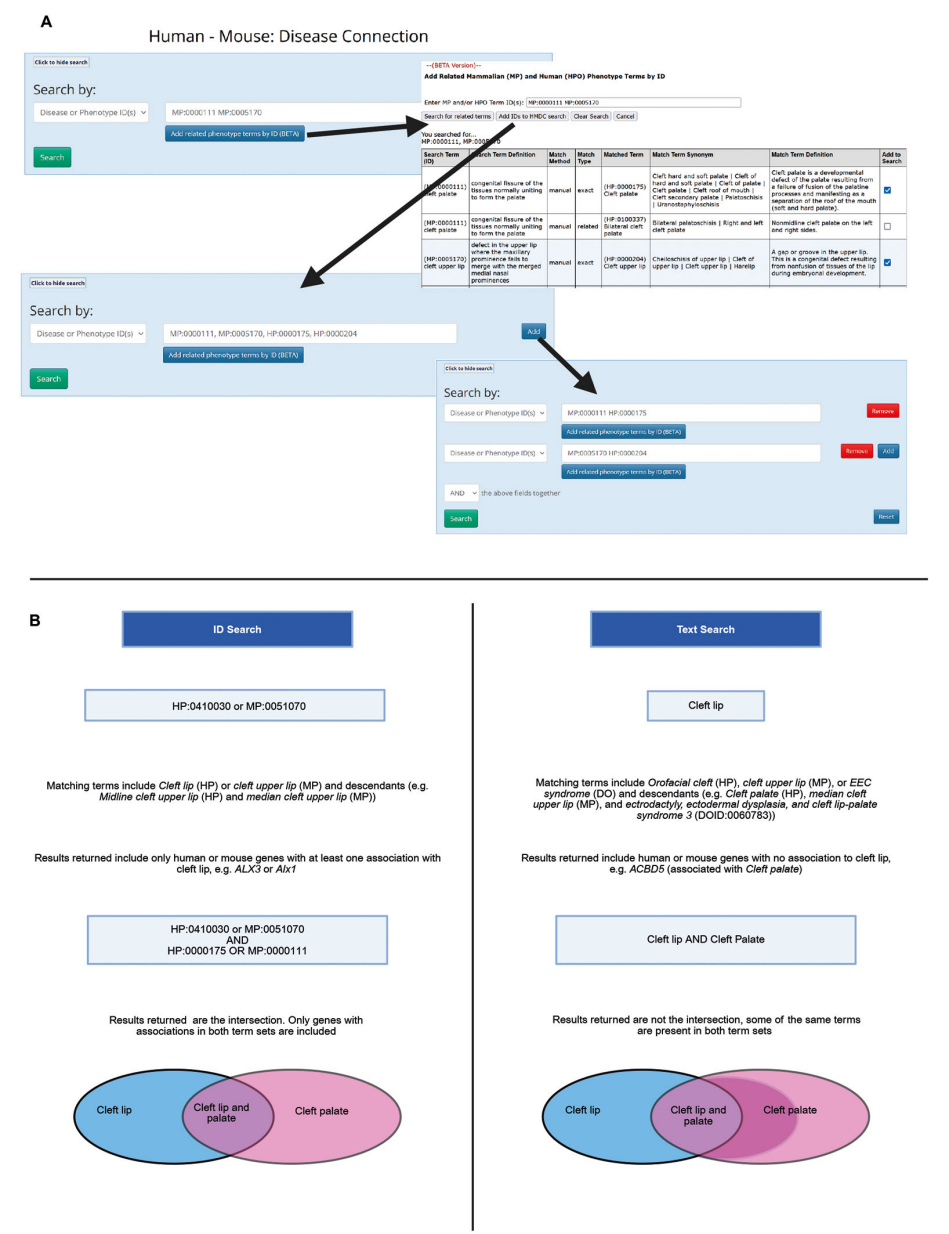
**Summary of Human Mouse Disease Connection (HMDC) text versus ID search.** (A) Search using the HMDC match tool. MP term IDs are entered in the HMDC disease ID search box. Then click the ‘Add related phenotype terms by ID’ button to find and select related HP term IDs and add these to the search. Finally, add the second search by ID box and move one set of terms to complete the search set up. (B) Searching the HMDC using IDs or text. The ID search returns only the exact term plus its descendants. In the search for Cleft lip AND Cleft palate using IDs returns only the intersection, only those genes with association to both terms. A text search for Cleft lip AND Cleft palate returns many results that are associated only with one of the terms (primarily cleft palate due to synonym matches for ‘Orofacial cleft’). Created in BioRender by Bello, S. (2025). https://BioRender.com/ag5h3l0. This figure was sublicensed under CC-BY 4.0 terms.

If, for example, a user wanted to find genes in mouse and human associated with cleft palate AND cleft lip they could start by searching the match tool for each of these term IDs (MP:0000111 and MP:0005170). This would return a list of matching HPO terms. The results include information about the match method and type, along with the full set of synonyms and definition for the matching term to aid the user in selecting terms. Checking the ‘Add to Search’ box adds the term(s) to a list for the HMDC search. Clicking ‘Add IDs to HMDC’ search then adds all the selected and searched for IDs to the HMDC search (Fig. 4A). To do the ‘AND’ search of cleft lip and cleft palate you would need to add a second search box and move one pair of MP and HPO IDs to the second box, as entering a list of IDs in a single search box does an ‘OR’ search between these IDs. Columns with matches to the searched term are highlighted in purple in the search results. Trying to do the same search using text search returns more results and highlights more columns. These increases are because of matches to terms outside the phenotype ontologies and to additional terms within the phenotype ontologies (Fig. 4B). This can also result in returning results that do not match the intent of the search. For example, the human gene *ACBD5* is returned by text search but not phenotype ID search. This gene is associated with the HPO term ‘Cleft palate’ but not ‘Cleft lip’. The text search returns this because the HPO term ‘Orofacial cleft’ (HP:0000202) has the synonym ‘Cleft lip, cleft palate’, and the text search looks for any term label or synonym that matches the text, then returns annotations to that term or any of its descendants. Because the HPO single term matches both of the text entries, the results will include any human gene associated with a single annotation to cleft lip or cleft palate rather than being limited to those with annotations to both.

Links from the MGI HPO browser automatically launch an HMDC ID search that returns only human results. The new MP-HP match tool makes it easy for a user to add matching or related MP terms to the search to compare between species. If a user selected ‘Short term memory impairment’ (HP:0033687) from the HPO browser, the initial set of results is limited to five human genes. By opening the search box and using the MP-HP match tool, they could add the related MP term ‘abnormal short-term spatial reference memory’ (MP:0008431) to expand the search to include mouse results.

## DISCUSSION

The utility of data from model organisms to advance the understanding of human disease is significantly enhanced by the ability to integrate and reason over these data. The focused expansion of the MP to more fully encompass the full spectrum of disease model phenotypes and improvements in the alignment with the HPO will facilitate the translation of annotated data and increase the reliability of tools relying on these data. The incorporation of the model data annotated with the MP into tools for variant prioritization has highlighted the translational value of these integrated data. The expansion to cover granular phenotypes in models within the hierarchy of the ontology allows researchers to lump or split data as desired and gain easy access to mechanistic insights drawn from the model data. Development driven by experts annotating real data will allow the MP to continue to grow and evolve to meet the needs of researchers, clinicians and bioinformaticians. We welcome feedback and contributions either through GitHub issues or by emailing us directly using the pheno@jax.org address.

## MATERIALS AND METHODS

### Ontology editing

The MP is maintained using Protege 5.6.1. Edits are made in the mp-edit.owl file either manually or using ROBOT (Jackson et al., 2019) (a tool to work with OBO ontologies) and spreadsheet templates for bulk addition of terms. The relationship between MP ID and MP term definition is stably maintained. If a term definition needs to be modified and that modification would change the meaning of the term such that data annotations for that term might be rendered invalid, then the term is deprecated and replaced with a new term. No MP IDs are deleted from the file; instead, the status is changed to ‘deprecated=true’, following GO community deprecation standards.

A modified Ontology Development Kit (ODK) (Matentzoglu et al., 2022b) pipeline using GitHub actions is used for building release files. Release files are produced in Web Ontology Language (OWL), OBO and JavaScript Object Notation (JSON) format. Release artifacts include the primary file ‘mp’ in the three formats, along with the other variations derived from the ODK pipeline (see https://github.com/INCATools/ontology-development-kit). Current release primary files in the three formats along with the file containing translations of MP terms (mp-international.owl) are available on the MGI ftp site (https://www.informatics.jax.org/downloads/reports/index.html#pheno) and on the MP GitHub site (https://github.com/mgijax/mammalian-phenotype-ontology). Prior releases are also available on the MP GitHub site. Each release is tagged with a version based on the date of the GitHub release. All files are released with a CC-BY 4.0 license. Version, license and developers are included in the metadata of the files.

The first release using the ODK pipeline was done on 13 August 2019. This is the earliest release file on GitHub.

GitHub actions are used to update imports of other ontologies used in the MP to logically define terms. This process runs monthly but can also be triggered manually as needed. GitHub actions are also used to run quality control checks at two points in the editing and release process. After every commit to the repository, a standard set of checks (from ODKfull v1.5) is performed. This checks for curation errors such as missing labels or duplicate logical definitions. Checks are also run during the release pipeline to look for errors in imports or other issues that may have been missed in the commit integration tests. As a final check, files are loaded into MGI before the release is made official to use the checks built into the MGI load pipeline. Once files are successfully loaded into MGI, the GitHub release is made official.

### Disease selection and paper triage

For the Gabriella Miller Kids First pediatric disease project, disease terms within the KFDRC with the highest usage (i.e. the number of unique sampleIDs in KFDRC associated with that disease) were selected for further review. These were then reviewed to identify those with significant additional literature available for curation within MGI. For each disease, keyword searches were done on the MGI corpus to extract the set of uncurated papers with at least one mouse allele. In addition, keyword searches were done in PubMed to find any papers not yet imported into MGI. The papers were then reviewed to find those that (1) contained novel mouse models or (2) contained novel phenotypes for existing mouse models. Papers containing models involving alleles of genes not currently associated with the disease in mice or humans were prioritized for curation. During the curation process, phenotypes without matching terms in the MP were documented and evaluated for inclusion in the MP. Phenotypes reported for multiple mouse models or matching existing terms in the HPO were given priority for addition to the MP. All GitHub issues related to this work were given the label Kids First.

Following the completion of paper curation, the full set of MP terms for mouse models of a given disease were extracted using MouseMine and manually mapped to HPO terms. Mappings were recorded in the SSSOM format (Matentzoglu et al., 2022a). The set of HPO terms associated with each disease was retrieved from the HPO website by using the ‘export associations’ function on the disease detail page(s) on the HPO site. Phenotypes were retrieved between April 2022 and April 2024. The HPO terms were then mapped to MP terms using SSSOM format. During mapping of HPO terms to MP terms, HPO terms with broad mammalian applicability were added to the MP.

For the COVID-19 project, publications reporting mouse models of COVID-19 or describing signs and symptoms of SARS-CoV-2 infection in human patients were collected. The set of signs and symptoms was seeded during the COVID-19 Biohackathon (https://github.com/virtual-biohackathons/covid-19-bh20) (Bello et al., 2021). The set from the workshop was expanded by extracting human signs and symptoms and mouse phenotypes from publications and mapped to MP and HPO terms using a modified SSSOM format. The SSSOM format had to be modified to allow for free text descriptions of signs and symptoms from the literature. Papers were primarily preprints tagged as COVID-19 SARS-CoV-2 preprints pulled from *bioRxiv* and *medRxiv* between June 2020 and March 2022. Issues were created in both the MP and HP GitHub repositories for potential new terms to add. In the MP GitHub repository, these issues were labeled with ‘COVID-19’.

For the Alzheimer's disease project, papers detailing specific phenotypes for patients and mouse models of Alzheimer's disease were retrieved from the OMIM website and keyword searches from PubMed and MGI. In addition, the clinical synopses from the OMIM website were reviewed for potential additional terms. Similar to the other disease projects, GitHub issues, labeled ‘AD project’, were created, and both new and existing Alzheimer's disease-relevant terms were mapped using the SSSOM standard.

Other areas (e.g. infertility, amino acid level, musculoskeletal, integument) of the MP were selected for revision and expansion based on MGI and community priorities. The same protocol was used for these, with a literature review followed by curation of phenotypes with concomitant addition of new terms and revision of relationships between existing terms.

### MP-HPO SSSOM mapping

Mappings were done manually and maintained in the SSSOM format. Metadata for each mapping include the ORCID iD(s) of the curator(s) responsible for the mapping. Match types are based on the skos mapping predicates: SKOS:exactMatch, SKOS:broadMatch, SKOS:narrowMatch, SKOS:relatedMatch, SKOS:closeMatch (https://www.w3.org/TR/skos-reference/#mapping). All mappings are made with the HP term as object and the MP term as the subject. This means that SKOS:broadMatch translates to the HPO term representing a broader class of phenotype compared to the MP term. In determining matches, the inherent restriction of all HPO terms to only humans was ignored. So, ‘Short femur’ (HP:0003097) was called an exact match to ‘short femur’ (MP:0003109), even though, technically, the HPO term only applies to humans, while the MP term applies to all mammals. Questions were noted in the mapping spreadsheet, and links to MP or HPO GitHub issues were included when these exist.

MP-HPO lexical matches are generated by MGI using an algorithm. Term and synonym labels are used to generate these mappings. Matches are only called when all the words in the label match across ontologies. Mappings are generated based on comparisons of term label-label, label-synonym and synonym-synonym pairs. An exact match is called when there is a match between term label-label, term label-exact synonym, or exact synonym-exact synonym. A narrow match is called when an MP narrow synonym matches an HPO term label or exact synonym. See  Table S1 to view the full matrix of match types. Lexical mappings are regenerated on a weekly basis, and the resulting mappings will be pushed in a SSSOM formatted file to the MHMI repository.

### MP-HPO mapping-based revisions

ROBOT was used to extract MP and HP terms mapped as ‘exact match’ along with their logical definitions. This was used to identify cases in which manual mapping called an MP and HP term an ‘exact match’, but the two terms had different logical definitions. Discrepancies were reviewed by MP and HPO developers to attempt to resolve the difference and agree on a single logical definition or revise the mapping, if it was decided the terms were not meant to represent the same phenotype. This same process was used to extract cases in which the MP and HP terms had the same logical definitions but had not been manually mapped as exact matches.

### Incorporation of uPheno patterns

The uPheno project has developed patterns for automated creation of phenotype terms including logical definitions (Matentzoglu et al., 2025). These patterns, along with spreadsheets containing terms from other ontologies, have been incorporated into the MP release pipeline to generate logical definitions for terms. The spreadsheets and patterns in use are stored in the MP GitHub repository. ROBOT was used to extract logical definitions for existing terms that appeared to match a pattern, and the logical definitions were placed into spreadsheets formatted for use with the patterns. Spreadsheets were reviewed to ensure that the terms fit each pattern. Patterns and the matching spreadsheets were then added to GitHub. Running the ‘refresh imports’ GitHub action replaces any existing logical definitions in the MP file with those generated by the pattern file and data spreadsheet.

### HMDC MP-HPO matching tool

The HMDC MP-HPO matching tool makes use of all of the MGI MP-HPO mappings as well as some additional mappings in the MHMI GitHub repository. Non-MGI-generated files from the repository included in the tool include all of the files generated by the IMPC. Only files that included calls for match type were imported for use in the tool. When a pair of terms is matched based on multiple methods, only a single match method is shown, with priority being manual>logical>lexical. When a pair of terms have more than one match type from the same method, only a single match type is shown, with the priority being exact>narrow>broad>related.

## Supplementary Material

10.1242/dmm.052385_sup1 Supplementary information
