# Supplementary material for: Expanding and refining the Mammalian Phenotype Ontology to enhance disease model discovery
Source: Dis Model Mech. 2025 Oct 28;18(10):dmm052385. doi: 10.1242/dmm.052385 (PMC12590472; doi:10.1242/dmm.052385)
Supplement: Supplementary information [file dmm-18-052385-s1.pdf]

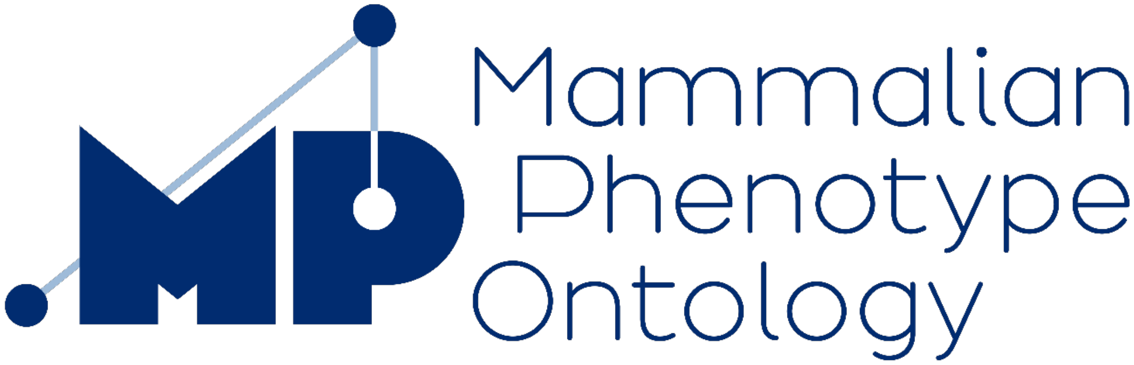

Fig. S1. Mammalian Phenotype Ontology logo. Created by the Jackson Laboratory creative team.

Table S1. Matrix of lexical matches. All matches were made from the perspective of the HPO term as the subject. Thus, narrow means the HPO term is narrower in meaning compared to the MP term. Some synonym-to-synonym matches were not made (nd) as the match type was too ambiguous or there were no examples of this type of match between the two ontologies for review.

	MP term label	MP exact synonym	MP narrow synonym	MP broad synonym	MP related synonym
HPO term label	exactMatch	exactMatch	narrowMatch	broadMatch	relatedMatch
HPO exact synonym	exactMatch	exactMatch	narrowMatch	broadMatch	relatedMatch
HPO narrow synonym	broadMatch	broadMatch	broadMatch[1]	nd[2]	nd[2]
HPO broad synonym	narrowMatch	narrowMatch	nd[2]	narrowMatch	nd[2]
HPO Related synonym	relatedMatch	relatedMatch	relatedMatch	nd[3]	nd[3]

[1]Two examples of this type were found. Both were determined to be a broadMatch
[2]No examples of this type found
[3]Review of the examples for this category gave incorrect results so this match was not made
